# Fluorinated Adenosine A_2A_ Receptor Antagonists Inspired by Preladenant as Potential Cancer Immunotherapeutics

**DOI:** 10.1155/2017/4852537

**Published:** 2017-10-19

**Authors:** Gengyang Yuan, Tanner C. Jankins, Christopher G. Patrick, Phaethon Philbrook, Olivia Sears, Stephen Hatfield, Michail Sitkovsky, Neil Vasdev, Steven H. Liang, Mary Jo Ondrechen, Michael P. Pollastri, Graham B. Jones

**Affiliations:** ^1^Department of Chemistry and Chemical Biology, Northeastern University, 360 Huntington Avenue, Boston, MA 02115, USA; ^2^New England Tissue Protection Institute, Northeastern University, 360 Huntington Avenue, Boston, MA 02115, USA; ^3^Gordon Center for Medical Imaging and Division of Nuclear Medicine and Molecular Imaging, Massachusetts General Hospital and Department of Radiology, Harvard Medical School, 55 Fruit St., Boston, MA 02114, USA; ^4^Clinical and Translational Science Institute, Tufts University, 800 Washington Street, Boston, MA 02111, USA

## Abstract

Antagonism of the adenosine A_2A_ receptor on T cells blocks the hypoxia-adenosinergic pathway to promote tumor rejection. Using an* in vivo* immunoassay based on the Concanavalin A mouse model, a series of A_2A_ antagonists were studied and identified preladenant as a potent lead compound for development. Molecular modeling was employed to assist drug design and subsequent synthesis of analogs and those of tozadenant, including fluorinated polyethylene glycol PEGylated derivatives. The efficacy of the analogs was evaluated using two* in vitro* functional bioassays, and compound** 29**, a fluorinated triethylene glycol derivative of preladenant, was confirmed as a potential immunotherapeutic agent.

## 1. Introduction

**Figure 1:**
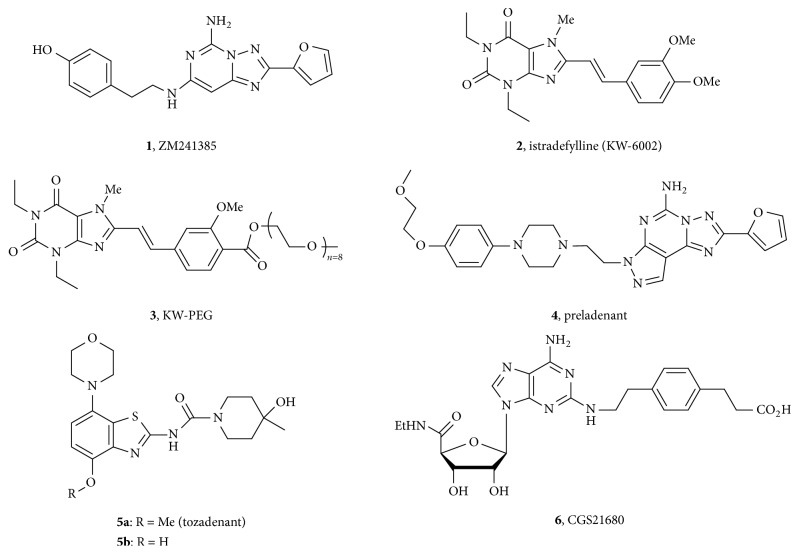
Structures of selected A_2A_R antagonists** 1**–**5a** and A_2A_R agonist CGS21680 (CGS,** 6**).

The adenosine receptors belong to the G protein-coupled receptor (GPCR) family including A_1_, A_2A_, A_2B_, and A_3_, four subtypes based on their different subcellular localization, signal transduction pathways, activation profiles, ligand binding profiles, and G protein binding preferences [1, 2]. Adenosine receptor coupling and subsequent dissociation with G_i_ and G_s_ proteins serve to regulate the level of adenylate cyclase activity, thus controlling levels of intracellular cAMP, a second messenger known to trigger a complex sequence of cellular events [1–3]. As a consequence, A_2A_R has become a drug discovery target of increased interest, implicated in diseases such as neurodegenerative disorders (e.g., Parkinson's disease), cardiac ischemia, inflammation, and cancer [4–6]. After over a decade of effort applied to xanthine based A_2A_R antagonists, a derivative KW-6002 (istradefylline,** 2**) was developed and approved in 2013 as an anti-Parkinson drug in Japan under the brand name Nouriast®. The molecule preladenant** (4)** completed Phase II clinical trials for Parkinson's disease but failed to show efficacy in subsequent Phase III trials. However, tozadenant (SYN115,** 5a**) entered Phase III trials in 2015 for the same indication (Figure 1) [7–9].

Given the surge in interest in A_2A_R antagonists, we have focused effort on the immunomodulatory capacity of agents. We have previously demonstrated antagonism of the hypoxia-adenosinergic pathway, wherein hypoxia-driven accumulation of extracellular adenosine triggered immune suppression via A_2A_R activation on the surface of immune cells [10–15]. Subsequent A_2A_R antagonism by ZM241385** (1)** led to delayed growth of CL8-1 melanoma in mice and increased levels of endogenous antitumor T cells [10–15]. Derivatization of xanthine** 2** led to a PEG derivative (KW-PEG,** 3**), which showed enhanced properties, including cAMP suppression and cytokine IFN-gamma restoration [16]. Spurred by these findings we were motivated to employ molecular modeling methods to design optimized derivatives (PEG) of other classes of A_2A_R antagonists and to explore both their immunomodulatory capacity and potential to be converted to functional imaging agents.

## 2. Materials and Methods

**Figure 2:**
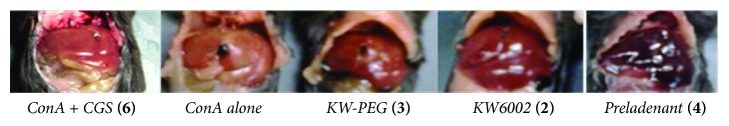
ConA-induced liver damage in C57BL/6 mice via CGS** (6)**,** 2**,** 3** and** 4**. Female C57BL/6 mice (*n* = 5) were first injected with A_2A_R agonist CGS (**6**, 2 mg kg^−1^), 2 mg kg^−1^** 2**,** 3**, and** 4** separately, and then Con A (20 mg kg^−1^). Con A-induced liver damage evaluated at 8 h.

To select lead compounds for immunotherapy application, an* in vivo* Concanavalin A (ConA)-induced liver damage assay was carried out in C57BL/6 mice through the pharmacological activation and deactivation of A_2A_Rs [17]. A variety of compounds were screened including** 2**,** 3,** and** 4** [10, 18, 19]. As shown in Figure 2, compound** 4** imparted the most severe immune induced liver damage and was selected as a core structure for analog design. Fluorinated analogs were envisioned to potentially serve as leads to ultimately be labeled at the distal position with fluorine-18 (*t*_1/2_ = 109.8 min), for diagnostic imaging with positron emission tomography (PET). A series of fluorinated PEG groups with increasing chain lengths were proposed for chemical modification to map the structure-activity relationship (SAR). Such modifications increase both hydrophilicity and molecular weight (MW), potentially to reduce blood-brain barrier (BBB) penetration as predicted by the central nervous system multiparameter optimization (MPO) score reported by Wager et al. [20].

**Figure 3:**
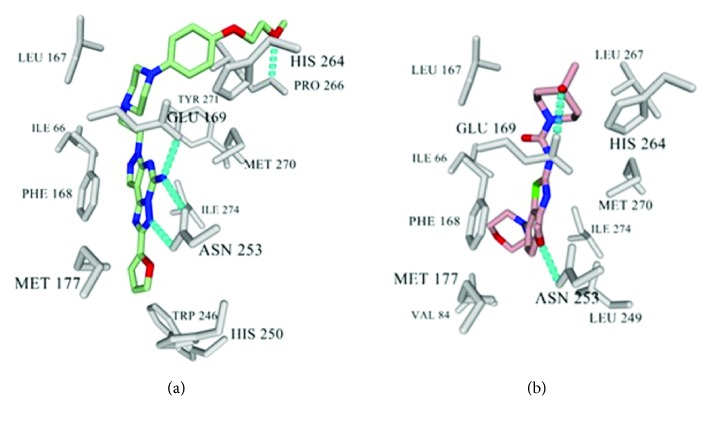
Glide XP docking results of** 4** and** 5a**. The interacting residues of A_2A_R are colored grey and the H-bond is represented as a dotted line. (a)** 4** and (b)** 5a** renderings from YASARA [39].

To locate the ideal position for PEG attachment, molecular modeling was utilized based on our previously constructed homology model, which derived from the crystal structure of A_2A_R in complex with** 1** (PDB ID 3EML) and includes a resolved EL2 cap [16, 21, 22]. This technique employed Glide (Schrödinger, version 10.4, LLC, New York, NY, 2015) extra precision (XP) docking to gain insights into the ligand-protein binding interactions [23–26]. As shown in Figure 3(a),** 4** almost occupies the entire binding pocket of A_2A_R and shares similar key binding interactions as known ligand** 1**. Noteworthily, the methoxyethyl ether group of** 4** projects into the cytosolic solution and forms an additional H-bond with Pro266 at the solvent-exposed surface of the A_2A_R, connecting with the cytosolic solution. Similarly, the current clinical candidate** 5a** also occupies a position near the edge of the A_2A_R binding pocket, where the piperidine quaternary alkyl group forms hydrophobic interactions with Leu267 and His264 and the tertiary alcohol group forms a hydrogen bond with Glu169 (Figure 3(b)) [27, 28]. It was thus suggested that introduction of hydrophilic and fluorinated PEG groups at the phenolic position of** 4** and the piperidine component of** 5a** would not impact key binding events of their core structures as the pendant groups would be capable of engaging in hydrogen bonds at the termini or in the case of their chains via hydrated networks. Accordingly, the octaethylene glycol monomethyl ether moiety, a tolerable substituent in prior studies on compound** 2** [16] in conjunction with the phenyl-piperazine linker inherent in** 4**, was introduced to** 5a** and syntheses designed. In addition, synthesis of a demethylated version of the compound** (5b)** was planned, as such could be a useful intermediate for radiotracer synthesis (as either [^11^C]**5a** via a one-step [^11^C]CH_3_I methylation or a base-promoted coupling with an ^18^F-labeled short alkyl chain) at this locus.

**Scheme 1:**
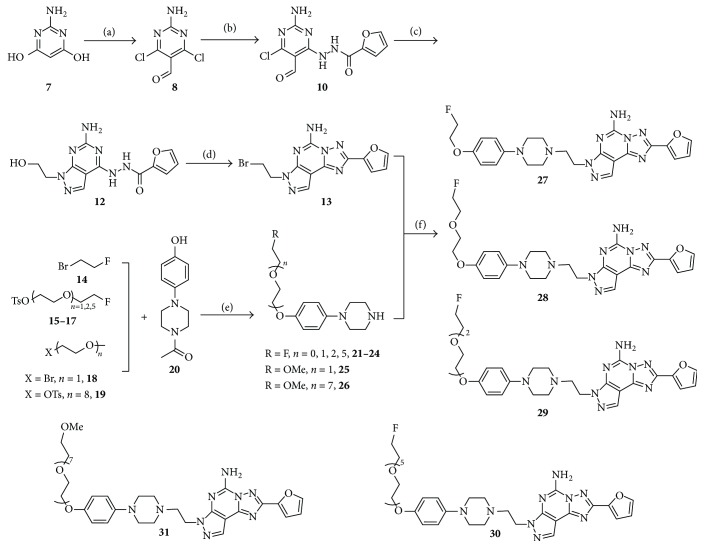
Synthesis of** 4** and its PEGylated analogs. Conditions: (a) POCl_3_, DMF, 78%; (b) 2-furoic acid hydrazide** (9)**, Na_2_CO_3_, MeCN; (c) 2-hydroxyethyl hydrazine** (11)**, 78% over 2 steps; (d) POCl_3_, ZnBr_2_, 45%; (e) (i) NaH, DMF; (ii) 6 N HCl. 67% (*n* = 0, 21), 62% (*n* = 1, 22), 56% (*n* = 2, 23), 35% (*n* = 5, 24), 70% (*n* = 1, 25), 70% (*n* = 7, 26); (f)* N,N*-diisopropylethylamine (DIPEA), DMF, 52% (*n* = 0,** 27**), 47% (*n* = 1,** 28**), 33% (*n* = 2,** 29**), 25% (*n* = 5,** 30**), 55% (*n* = 1,** 4**), and 25% (*n* = 7,** 31**).

Synthesis of reference compounds** 2** and** 3** was performed using refinements of reported methods which produced superior yields independent of scale [16]. For example, use of a mild (AIBN/NBS promoted) route to the 8-substituted xanthine scaffold resulted in an improvement in yield from 22% to 56% for this key step (see experimental section) [29]. Compounds** 4** and** 5a** were synthesized based on modified literature methods (Scheme 1) [30–33], key intermediate** 13** obtained from compound** 7** via Vilsmeier-promoted halogenation and formylation, one-pot cascade condensation with 2-furoic acid hydrazine** (9)** and 2-hydroxyethyl hydrazine** (11)**, Dimroth rearrangement to effect triazole formation, and finally bromination with POCl_3_/ZnBr_2_. The piperazine components were prepared starting from either commercially available fragment** 14** or fluorination/activation of the known mono- or ditosylated PEG chains** (15**–**19)** and subsequent coupling reaction with 1-(4-(4-hydroxyphenyl) piperazin-1-yl)ethan-1-one** (20)** and then deacetylation prior to the final coupling reaction with intermediate** 13** to furnish** 4** and the desired analogs** 27**–**31** [30, 34].

**Scheme 2:**
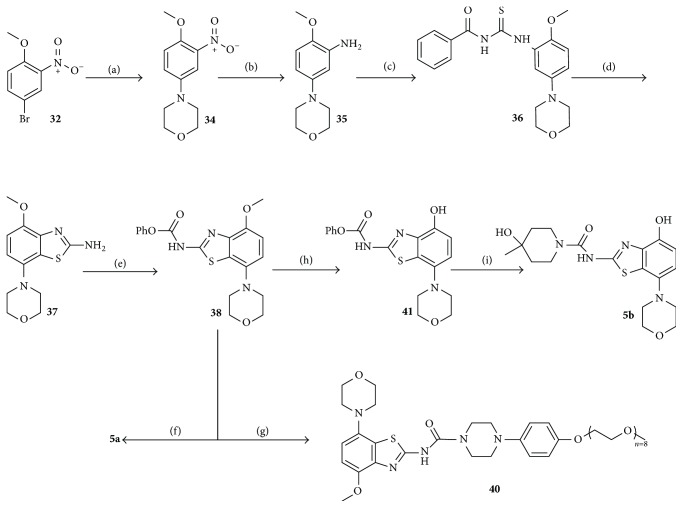
Synthesis of** 5a**,** 5b**, and PEGylated analog** 40**. Conditions: (a) morpholine** (33)**, K_3_PO_4_, 2-biphenyl-dicyclohexylphosphine, Pd(OAc)_2_, dimethoxyethane, 37%; (b) Sn powder, EtOH/con.HCl, 66%; (c) benzoyl isothiocyanate, acetone, 99%; (d) (i) NaOMe, MeOH; (ii) Br_2_, CHCl_3_, 73%; (e) phenyl carbonochloridate, pyridine, dichloromethane, 94%; (f) 4-methylpiperidin-4-ol hydrochloride** (39)**, DIPEA, THF, CHCl_3_, 53%; (g)** 26**, DIPEA, THF, CHCl_3_, 28%; (h) BBr_3_, dichloromethane, 52%; (i)** (39)**, DIPEA, THF, CHCl_3_, 62%.

Synthesis of** 5a** is illustrated in Scheme 2. The bromide** 32** was subjected to palladium-catalyzed coupling with morpholine** (33)**, stannous nitro reduction, condensation with benzoyl isothiocyanate, bromine promoted formation of the benzothiazole ring, and installation of the piperidine ring through intermediate** 38**. Preparation of analog** 40** was achieved via coupling of** 26** and** 38** [31]. Direct demethylation of** 5a** with BBr_3_ did not lead to the desired product** 5b** but instead led to decomposition and bromination of the tertiary alcohol [34]. Likewise, l-selectride promoted demethylation of** 5a** led to very poor yield of product** 5b** (5%) [35]. The sequence was finally realized when the phenyl carbamate protecting group of** 38** was employed. With demethylation achieved, the phenyl carbamate protecting group (of** 41**) was replaced by 4-methylpiperidin-4-ol** 39** to afford desmethyl tozadenant,** 5b**. Full details of all experimental procedures, bioassays, and molecular modeling are described in the Supplementary Material available online at https://doi.org/10.1155/2017/4852537.

## 3. Results and Discussion

**Figure 4:**
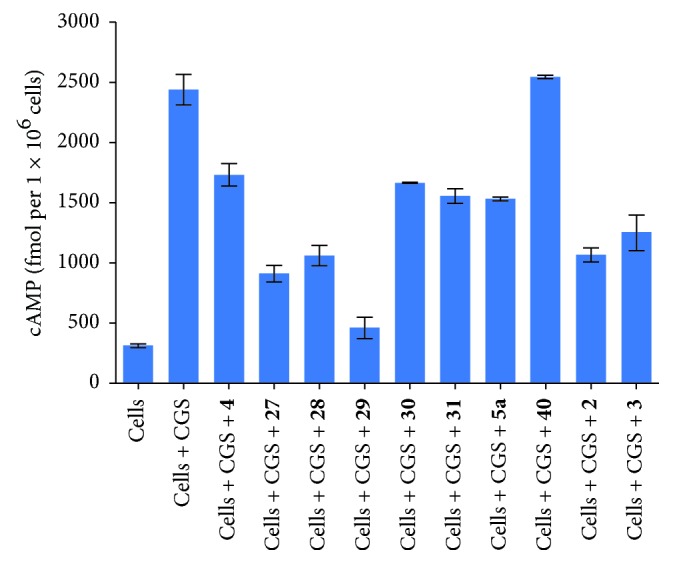
Intracellular cAMP levels in lymphocytes after incubation with vehicle, 1 *µ*M CGS, and 1 *µ*M CGS plus 1 *μ*M of compounds** 4** (preladenant),** 27**–**31**,** 5a** (tozadenant),** 40**,** 2** (KW-6002), and** 3** (KW-PEG) are shown. The intracellular cAMP levels were determined 15 min following stimulation using quantitative cAMP ELISA and are expressed as fmol/10^6^ cells. Data shown represent mean ± SEM of triplicate samples.

Bioassay of compounds** 27**–**31** and** 40** and their parent compounds (**4** and** 5a**) was conducted using two functional assays that evaluate A_2A_R binding-dependent signaling through A_2A_R on the surface of T cells [16]. The first assay screens compounds on the basis of their extent of inhibition of A_2A_R-induced intracellular cAMP accumulation in A_2A_R expressing lymphocytes [36, 37]. The A_2A_R agonist, CGS21680 (CGS,** 6**), was used to activate A_2A_R. As shown in Figure 4, all of the above compounds, except** 40**, were able to prevent CGS-mediated signaling. Stronger antagonism was observed for the preladenant-based analogs** 27**–**29** versus the previously evaluated compounds** 2** and** 3**. Further increments of the PEG chain length resulted in decreased antagonism (compounds** 30** and** 31**). Surprisingly,** 5a** showed inferior antagonism to that of compounds** 2** and** 3**, and its derivative** 40** exhibited no suppression of intracellular cAMP accumulation.

**Figure 5:**
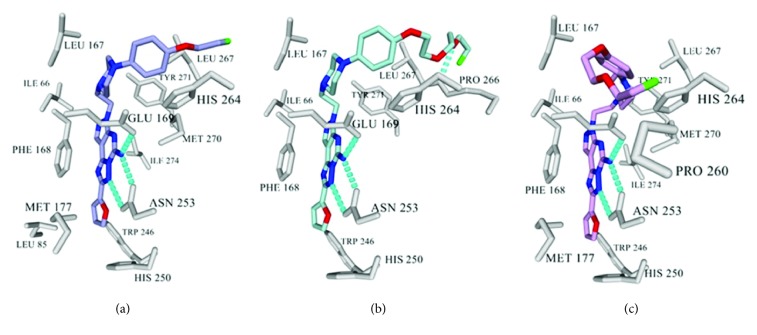
Docking results of** 27**–**29** via Glide XP method. The interacting residues of A_2A_R are colored grey and the H-bond is represented as cyan dotted line. (a) Compound** 27.** (b) Compound** 28.** (c) Compound** 29**. Rendered from YASARA [39].

An evaluation of the positive hits in the cAMP assay** (27**–**29)** was carried out in silico by Glide docking to study their binding orientation in A_2A_R. The docking results confirmed the initial assumption for such analog design (Figure 5), the core structures of** 27**–**29** anchoring in similar positions as** 4**, forming identical key binding interactions with Asn253, Glu169, and Phe168. The installed PEG chains interact with the residues at the edge of A_2A_R via hydrophobic and H-bonding interactions.

**Figure 6:**
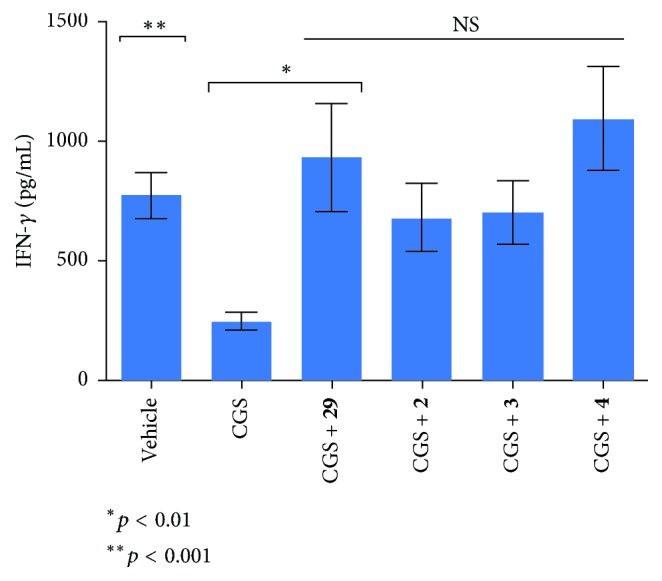
The IFN-gamma production by splenocytes after activation with 0.1 *µ*g/mL anti-CD3 and when treated with vehicle, 1 *µ*M CGS, and 1 *µ*M CGS plus 1 *µ*M compounds** 29**,** 2** (KW-6002),** 3** (KW-PEG), and** 4** (preladenant) separately is shown. The IFN-gamma levels were determined in the supernatant one day following stimulation using quantitative ELISA and are expressed as pg/mL. Data shown represent mean ± SEM of triplicate samples.

The second immunoassay assesses secretion of the cytokine IFN-gamma, since it is considered to be sensitive to the A_2A_R signaling pathway [16]. In these assays, during T cell receptor (TCR) activation by the CD3 ligand, C57BL/6 mice splenocytes T cells are incubated with A_2A_R agonist CGS to inhibit IFN-gamma secretion resulting from A_2A_R-induced immunosuppression via intracellular cAMP. Effective A_2A_R antagonists block the A_2A_R-activated signal, thus restoring secretion of the cytokine to potentiate and prolong the immune response. Compounds** 29**,** 2**,** 3**, and** 4** were evaluated (Figure 6), and compound** 29** showed similar capacity to that of** 4**, both of which resulted in superior restoration of IFN-gamma secretion compared to either** 2** or** 3**.

Given the promising results in functional assays, the physicochemical properties of compound** 29** and its homologs were determined, including its log⁡*D*_7.4_, aqueous solubility, human plasma protein binding (PPB), and metabolic stability [human liver microsome and rat hepatocyte clearance] as shown in Table 1. Broadly similar results were obtained, principle differences being enhanced aqueous solubility for** 27**, whereas intrinsic clearance was superior for** 29** in the rodent derived line and for** 28** in the human cell line. Reduced clearance for** 27** in turn may bode well for use in biodistribution studies [38].

## 4. Conclusions

In summary, we have designed and synthesized a family of PEGylated analogs of** 4** and** 5a** using molecular modeling techniques. Lead compound** 29**, a fluorinated triethylene glycol derivative of preladenant, was identified, which shows promising results in two functional immunoassays and physicochemical assays. Future work will focus on detailed mechanistic studies on the mode of action of** 29** and investigation of its use as a potential cancer immunotherapeutic agent.

## Supplementary Material

Experimental procedures for chemical synthesis and evaluation of compounds.

## Figures and Tables

**Table 1:** Physicochemical properties and docking results of compounds **27**–**29**.

Compound	Glide score	log⁡*D*_7.4_	Aqueous solubility (*μ*M)	Human PPB (%)	HLM CLint (*μ*L/min/mg)	Rat hepatocyte CLint (*μ*L/min/10^6^)
**27**	−12.2	2.8	74	99	16.5	29.9
**28**	−11.8	2.5	2	98.7	71.9	12.8
**29**	−12.3	2.3	10	98.3	66.1	72.9
