# Supplementary material for: Fluorinated Adenosine A2A Receptor Antagonists Inspired by Preladenant as Potential Cancer Immunotherapeutics
Source: Int J Med Chem. 2017 Oct 19;2017:4852537. doi: 10.1155/2017/4852537 (PMC5671725; doi:10.1155/2017/4852537)
Supplement: Supplementary file 1 — Experimental procedures for chemical synthesis and evaluation of compounds. [file 4852537.f1.pdf]

Fluorinated adenosine A_{2A} receptor antagonists inspired by Preladenant as potential cancer immunotherapeutics

Gengyang Yuan [a], Tanner C. Jankins [a], Christopher G. Patrick Jr.[a], Phaethon Philbrook [b], Olivia Sears [b], Stephen Hatfield [b], Michail Sitkovsky [b], Neil Vasdev [c], Steven H. Liang [c], Mary Jo Ondrechen [a], Michael P. Pollastri [a], Graham B. Jones [a,d*]

[a]Department of Chemistry and Chemical Biology, Northeastern University, 360 Huntington Avenue, Boston, MA, 02115 [b]New England Tissue Protection Institute, Northeastern University, 360 Huntington Avenue, Boston, MA, 02115 [c]Gordon Center for Medical Imaging & Division of Nuclear Medicine and Molecular Imaging, Massachusetts General Hospital and Department of Radiology, Harvard Medical School, 55 Fruit St., Boston, MA, 02114 [d]Clinical and Translational Science Institute, Tufts University Medical Center, 800 Washington Street, Boston, MA, 02111

EXPERIMENTAL PROCEDURES

All solvents were of reagent or anhydrous grade quality and purchased from Sigma-Aldrich, Alfa Aesar, or Fisher Scientific. All reagents were purchased from Sigma-Aldrich, Alfa Aesar, Fisher Scientific, or Oakwood Chemical, unless otherwise stated. All deuterated solvents were purchased from Cambridge isotopes. Analytical thin-layer chromatography (TLC) was performed on pre-coated glass-backed plates (EMD TLC Silica gel 60 F_{254}) and visualized using a UV lamp (254 nm), potassium permanganate stain. Silica gel for manual flash chromatography was high purity grade 40-63 μm pore size and purchased from Sigma-Aldrich. Yields refer to purified and spectroscopically pure compounds. NMR spectra were obtained with Bruker or Varian instruments, operating at 300 or 400 MHz respectively for ^1H acquisitions as noted. LCMS analysis was performed using a Waters Alliance reverse-phase HPLC, with single wavelength UV-visible detector and an LCT Premier time-of-flight mass spectrometer (electrospray ionization).

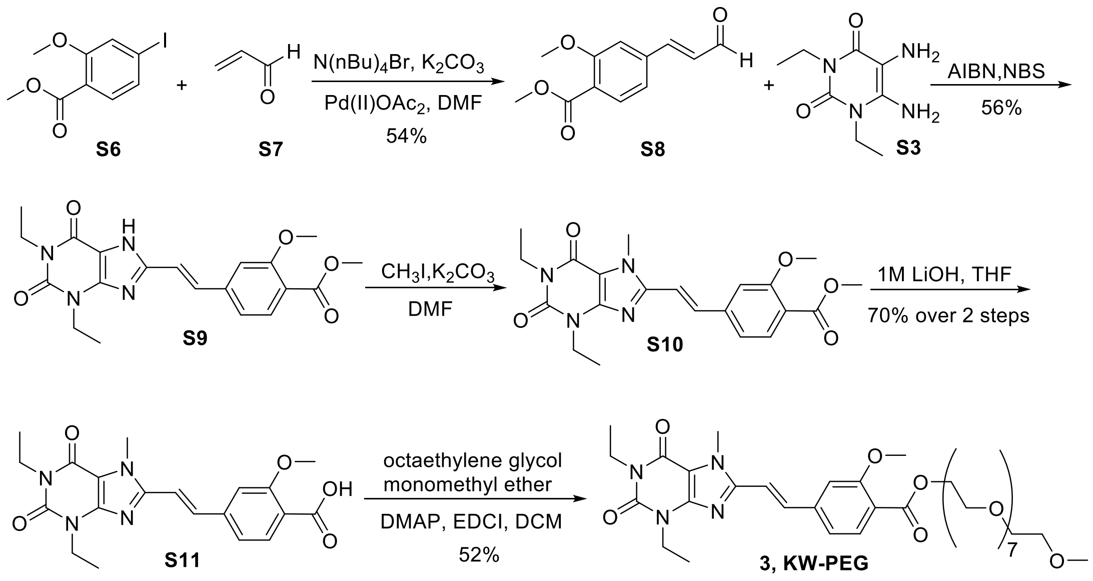

Modified synthesis of KW-PEG (**3**).

Synthesis of KW-PEG **3** was conducted using a reported method [6] with an improved modification for the synthesis of intermediate compound **S9** as follows:

*Synthesis of methyl (E)-4-(2-(1,3-diethyl-2,6-dioxo-2,3,6,7-tetrahydro-1H-purin-8-yl)vinyl)-2-methoxybenzoate (**S9**).* In a 500 mL round-bottom flask, the solution of 1,3-diethyl-5,6-aminouracil **S3** (3.958 g, 19.84 mmol) and (*E*)-methyl 2-methoxy-4-(3-oxoprop-1-en-1-yl)benzoate **S8** (4.369 g, 19.84 mmol) in acetonitrile / water (78 mL in a ratio of 9:1) was stirred for 15 min. To this mixture, a catalytic amount of AIBN (0.065 g, 2% mmol) was added and after stirring for 5 min, NBS was added in two portions (1.776 g and 0.71 g after 30 min (2.486g, 13.97mmol in total)). The reaction was stirred at room temperature for another 2.5 h to complete. The resulting precipitate was filtered under vacuum and purified by flash column chromatography using (1:1 ethyl acetate : hexane) to yield **25** as a yellow solid **S9** (4.420 g, 56%).

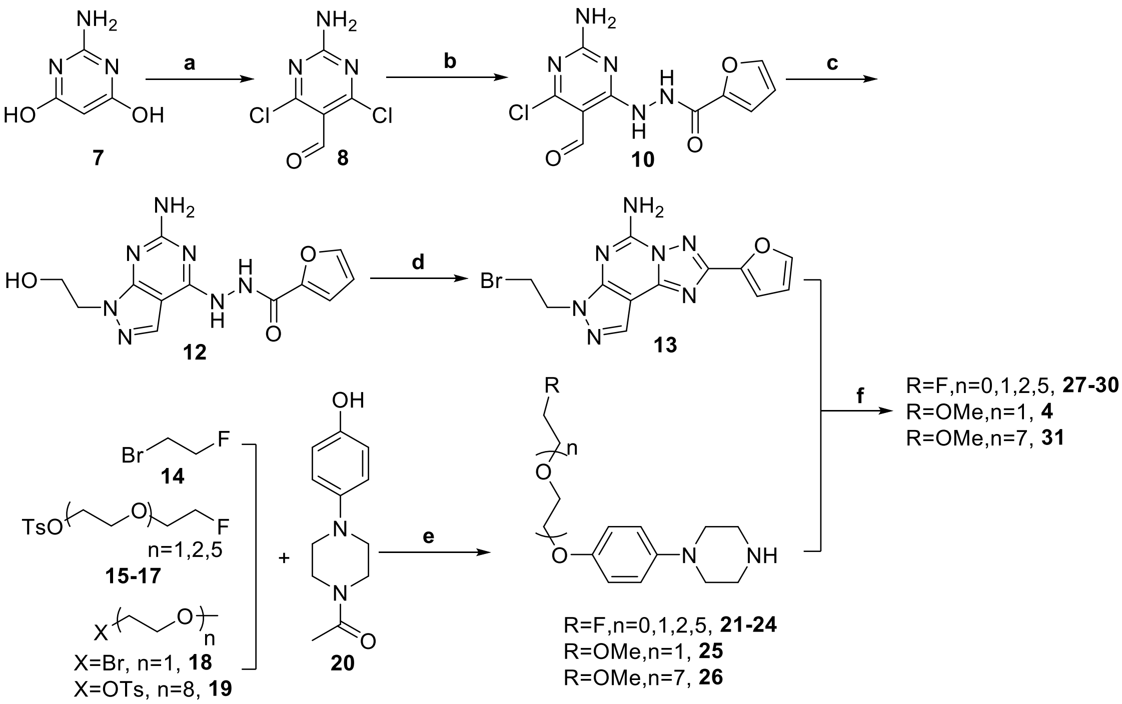

Scheme 1. Synthesis of **4** and its PEGylated analogs. Conditions: (a) $POCl_3$, DMF, 78%; (b) 2-furoic acid hydrazide (**9**), Na_2CO_3, MeCN; (c) 2-hydroxyethyl hydrazine (**11**), 78% over 2 steps; (d) $POCl_3$, $ZnBr_2$, 45%. (e) i. NaH, DMF; ii. 6NHCl. 67% (n = 0, 21), 62% (n = 1, 22), 56% (n = 2, 23), 35% (n = 5, 24), 70% (n = 1, 25), 70% (n = 7, 26); (f) *N,N*- Diisopropylethylamine (DIPEA), DMF, 52% (n = 0, 27), 47% (n = 1, 28), 33% (n = 2, 29), 25% (n = 5, 30), 55% (n = 1, 4), 25% (n = 7, 31).

Compounds **13** and **4** were prepared by literature methods.[15a] Spectroscopic data of compounds **7-13**, **15-17**, **19**, **21-22**, **25**, **4**, and **27-28** matched those reported in the literature.[15d]

Synthesis of 1-(4-(2-(2-(2-fluoroethoxy)ethoxy)ethoxy)phenyl)piperazine (23).[15a] To NaH (0.128 g, 5.34 mmol) in DMF (6.85 mL) was added the phenol **20** (0.784 g, 3.56 mmol), followed by compound **16** (1.2 g, 3.92 mmol). The mixture was stirred at room temperature for 18 h, concentrated, then partitioned between ethyl acetate and 5% citric acid. The organic layer was washed with 1M NaOH solution, then brine, dried over $MgSO_4$, and concentrated under vacuum to obtain a yellow oil. This material was heated to reflux in 6M HCl (4.3 mL) for 1 h. After cooling to ambient temperature the solution was basified to pH=10 (NaOH), extracted with CH_2Cl_2 and the organic extracts washed with water and then brine. The extracts were dried with $MgSO_4$ and concentrated *in vacuo* to give dark red oil, which was purified via flash column chromatography using a gradient of 1-5% MeOH in dichloromethane to yield the desired product

23 as yellow solid (0.62 g, 56%). ^{1}HNMR (400 MHz, $CDCl_3$) δ 6.84-6.90 (m, 4H), 5.30 (brs, 1H), 4.57 (dt, 2H, *J*= 47.6 Hz, 4.4 Hz), 4.09 (t, 2H, *J*= 5.1 Hz), 3.84 (t, 2H, *J*= 5.1 Hz), 3.80 (t, 1H, *J*= 4.4 Hz), 3.69-3.77 (m, 5H), 3.03-3.05 (m, 8H). ^{13}CNMR (100 MHz, $CDCl_3$) δ 153.1, 146.6, 118.3, 115.5, 83.3(d, *J*= 168.6 Hz), 71.0, 70.7, 70.5, 70.1, 68.0, 51.9, 46.4. LCMS found 313.2 $[M + H]^+$.

Synthesis of 1-(4-((17-fluoro-3,6,9,12,15-pentaoxaheptadecyl)oxy)phenyl)piperazine ***(24)****.*Following the same method for the synthesis of compound **23**, compound **24** was obtained as a yellow oil (0.11 g, 35%). ^{1}HNMR (400 MHz, $CDCl_3$) δ 6.83-6.88 (m, 4H), 5.30 (brs, 1H), 4.54 (dt, 2H, *J*= 48.4 Hz, 4.4 Hz), 4.07 (t, 2H, *J*= 5.9 Hz), 3.81 (t, 2H, *J*= 5.1 Hz), 3.76 (t, 1H, *J*= 3.7 Hz), 3.61-3.72 (m, 17H), 3.01-3.03 (m, 8H). ^{13}CNMR (100 MHz, $CDCl_3$) δ 151.8, 145.3, 117.0, 114.2, 82.0(d, *J*= 169.4 Hz), 69.7, 69.6, 69.5, 69.4, 69.3, 69.2, 68.8, 66.8, 50.7, 45.2. LCMS found 445.3 $[M + H]^+$.

Synthesis of 1-(4-((2,5,8,11,14,17,20,23-octaoxapentacosan-25-yl)oxy)phenyl)piperazine ***(26)****.*Following the same method for the synthesis of compound **23**, compound **26** was obtained as a yellow oil (0.78 g, 70%). ^{1}HNMR (400 MHz, $CDCl_3$) δ 6.83-6.88 (m, 4H), 5.30 (brs, 1H), 4.08 (t, 2H, *J*= 3.7 Hz), 3.83 (t, 2H, *J*= 5.1 Hz), 3.70-3.73 (m, 2H), 3.62-3.67 (m, 23H), 3.52-3.56 (m, 3H), 3.38 (s, 3H), 3.0-3.05 (m, 8H). ^{13}CNMR (100 MHz, $CDCl_3$) δ 153.0, 146.5, 118.2, 115.4, 72.0, 70.9, 70.7, 70.6, 70.0, 68.0, 59.1, 53.8, 51.9, 46.4. LCMS found 545.3$[M + H]^+$.

Synthesis of 7-(2-(4-(4-(2-(2-(2-fluoroethoxy)ethoxy)ethoxy)phenyl)piperazin-1-yl)ethyl)-2-(furan-2-yl)-7H-pyrazolo[4,3-e][1,2,4]triazolo[1,5-c]pyrimidin-5-amine ***(29)****.*[3] In a flame dried 10 mL round-bottom flask, compound **13** (0.204g, 0.58 mmol) and **23** (0.15 g, 0.48 mmol) were dissolved in 1.5 mL DMF, followed by the addition of DIPEA (0.38 mL). The reaction mixture was heated to 80 °C for 10 h. The mixture was diluted with water (5 mL), extracted with ethyl acetate, and the organic extracts washed with water and then brine and dried over $MgSO_4$ then concentrated in vacuo to produce a dark brown oil, which was purified via flash column chromatography using a gradient of 1-10% MeOH in dichloromethane to yield the desired product **29** as yellow powder (91 mg, 33%). m. p: 171-172°C. ^{1}HNMR (300 MHz, $CDCl_3$) δ 8.20 (s, 1H), 7.59-7.61 (m, 1H), 7.24 (d, 1H, *J*=3.5 Hz), 6.84-6.86 (m, 4H), 6.57-6.60 (m, 1H), 6.11 (brs, 2H), 4.62-4.65 (m, 1H), 4.47-4.55 (m, 3H), 4.08 (t, 2H, *J*=4.8 Hz), 3.79-3.85 (m, 3H), 3.69-3.75 (m, 5H), 3.06-3.09 (t, 4H, *J*=4.7 Hz), 2.97 (t, 2H, *J*=7.0 Hz), 2.73 (t, 4H, *J*=4.8 Hz).

^{13}CNMR (75 MHz, $CDCl_3$) δ 156.6, 152.9, 149.2, 148.1, 145.8, 145.6, 145.0, 144.5, 132.1, 117.9, 115.4, 112.5, 111.9, 97.3, 83.1 (d, *J*=169.2 Hz), 70.8, 70.5, 70.3, 69.9, 67.9, 56.9, 53.2, 50.4, 45.0. LCMS found 580.3 $[M + H]^+$.

*Synthesis of 7-(2-(4-(4-((17-fluoro-3,6,9,12,15-pentaoxaheptadecyl)oxy)phenyl)piperazin-1-yl)ethyl)-2-(furan-2-yl)-7H-pyrazolo[4,3-e][1,2,4]triazolo[1,5-c]pyrimidin-5-amine (**30**).* Following the same method for the synthesis of compound **29**, compound **30** was obtained as a tacky yellow solid (42 mg, 25%). ^{1}HNMR (400 MHz, d_6-DMSO) δ 8.16 (s, 1H), 8.05 (brs, 2H), 7.93 (s, 1H), 7.21 (d, 1H, *J*=2.9 Hz), 6.90 (d, 1H, *J*=8.8 Hz), 6.76-6.84 (m, 4H), 6.70-6.73 (m, 1H), 4.53-4.56 (m, 1H), 4.38-4.41 (m, 3H), 3.95-4.0 (m, 3H), 3.63-3.70 (m, 4H), 3.45-3.60 (m, 15H), 3.0 (t, 1H, *J*=5.1 Hz), 2.90-2.95 (m, 4H), 2.81 (t, 2H, *J*=5.9 Hz), 2.55-2.29 (m, 3H). ^{13}CNMR (75 MHz, d_6-DMSO) δ 156.6, 152.9, 149.2, 148.1, 145.8, 145.6, 145.0, 144.5, 132.1, 117.9, 115.4, 112.5,111.9, 97.3, 83.5 (d, *J*=165.8 Hz), 70.8, 70.5, 70.3, 69.9, 67.9, 56.9, 53.2, 50.4, 45.0. LCMS found 712.3 $[M + H]^+$.

*Synthesis of 7-(2-(4-(4-((2,5,8,11,14,17,20,23-octaoxapentacosan-25-yl)oxy)phenyl)piperazin-1-yl)ethyl)-2-(furan-2-yl)-7H-pyrazolo[4,3-e][1,2,4]triazolo[1,5-c]pyrimidin-5-amine (**31**).* Following the same method for the synthesis of compound **29**, compound **31** was obtained as a tacky yellow solid (0.15 g, 25%). ^{1}HNMR (300 MHz, $CDCl_3$) δ 8.16 (s, 1H), 7.54-7.55 (m, 1H), 7.20 (d, 1H, *J*= 3.5 Hz), 6.78-6.86 (m, 4H), 6.67 (brs, 2H), 6.56 (dd, 1H, *J*= 3.4, 1.8 Hz), 4.64 (t, 2H, *J*= 6.6 Hz), 4.06 (t, 2H, *J*= 4.8 Hz), 3.81 (t, 2H, *J*=4.8 Hz), 3.63-3.64 (m, 28H), 3.52-3.55 (m, 2H), 3.37 (s, 3H), 3.19-3.22 (m, 4H), 2.91-2.94 (m, 4H). ^{13}CNMR (75 MHz, $CDCl_3$) δ 156.5, 153.4, 149.0, 148.3, 145.6, 145.5, 144.8, 144.5, 132.4, 118.4, 115.4, 112.5, 111.9, 97.2, 71.8, 70.7, 70.6, 70.5, 70.4, 70.3, 69.8, 67.8, 59.0, 56.3, 54.0, 52.9, 49.6, 44.0, 42.2. LCMS found 812.4 $[M + H]^+$.

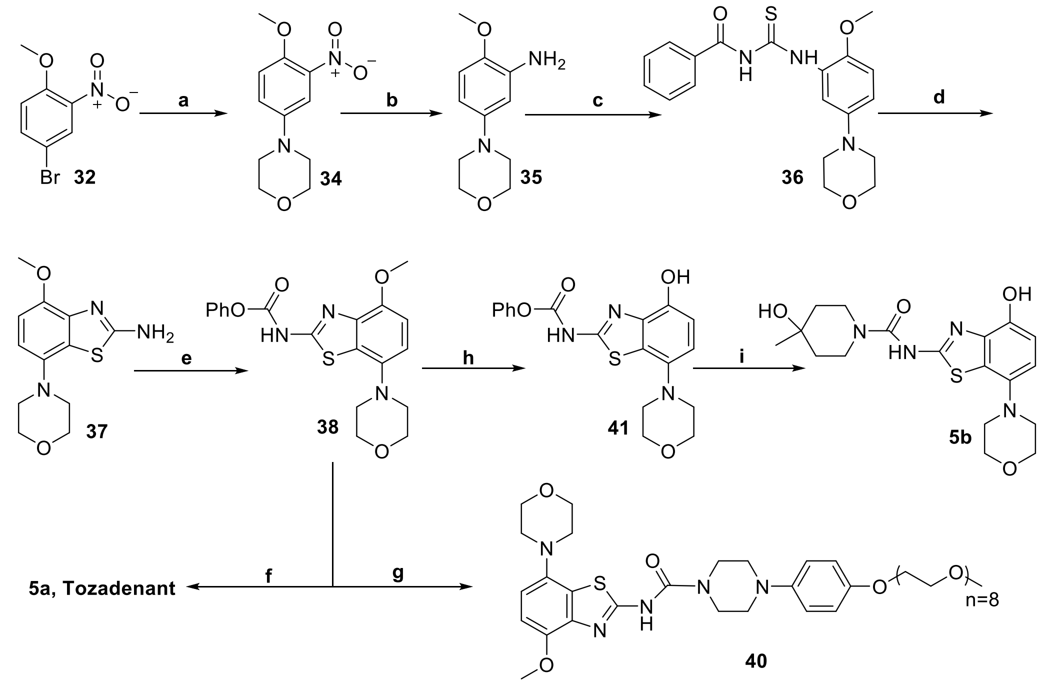

Scheme 2. Synthesis of **5a**, **5b** and PEGylated analog **40**. Conditions: (a) morpholine (**33**), K_3PO_4, 2-biphenyl-dicyclohexylphosphine, $Pd(OAc)_2$, dimethoxyethane, 37%; (b) Sn powder, EtOH/conc.HCl, 66%; (c) benzoyl isothiocyanate, acetone, 99%; (d) i. NaOMe, MeOH; ii. Br_2, $CHCl_3$, 73%; (e) phenyl carbonochloridate, pyridine, dichloromethane, 94%; (f) 4-methylpiperidin-4-ol hydrochloride (**39**), DIPEA, THF, $CHCl_3$, 53%; (g) **26**, DIPEA, THF, $CHCl_3$, 28%; (h) BBr_3, dichloromethane, 52%; (i) **39**, DIPEA, THF, $CHCl_3$, 62%.

*Synthesis of 4-(4-((2,5,8,11,14,17,20,23-octaoxapentacosan-25-yl)oxy)phenyl)-N-(4-methoxy-7-morpholinobenzo[d]thiazol-2-yl)piperazine-1-carboxamide (**40**).*[15b] To a solution of compound **38** (35.4 mg, 91.8 µmol) and *N,N*-Diisopropylethylamine (DIPEA, 48.7µL, 276.7 µmol) in chloroform (0.55 mL) was added a solution of compound **26** (50 mg, 91.8 µmol) in chloroform (0.33 mL) and tetrahydrofuran (0.33 mL). The resulting mixture was heated to reflux for 1 h. The reaction mixture was then cooled to ambient temperature and extracted with saturated aqueous sodium carbonate (0.17 mL) and water (2 x 0.1 mL). The solution was dried ($MgSO_4$), concentrated in vacuo, and the residue purified by flash column chromatography using a gradient of 1-3% MeOH in dichloromethane to produce compound **40** as a tacky pale yellow solid (21 mg, 28%). ^{1}HNMR (400 MHz, $CDCl_3$) δ 6.76-6.86 (m, 6H), 4.06 (t, 2H, *J*= 4.4 Hz), 3.90 (s, 3H), 3.86 (t, 4H, *J*= 4.4 Hz), 3.81 (t, 2H, *J*= 4.4 Hz), 3.67-3.75 (m, 6H), 3.56-3.66 (m, 24H), 3.53 (t, 2H, *J*= 4.4 Hz), 3.36 (s, 3H), 3.02-3.11 (m, 8H). ^{13}CNMR (100 MHz, $CDCl_3$) δ

153.8, 145.5, 140.7, 126.4, 119.0, 115.6, 112.5, 107.4, 72.1, 71.0, 70.9, 70.8, 70.7, 70.0, 68.0, 67.6, 59.3, 56.2, 52.0, 50.9, 44.3, 29.9. LCMS found 836.4 $[M + H]^+$.

*Synthesis of phenyl (4-hydroxy-7-morpholinobenzo[d]thiazol-2-yl)carbamate (**41**).*[16] To a solution of compound **38** (0.6 g, 1.48 mmol) in dichloromethane (31.2 mL) was added BBr_3 (10.6 mL, 1 M solution in dichloromethane) dropwise at -78 °C. The mixture was allowed to warm to room temperature and stirred overnight. Water (20 mL) was added to the mixture followed by NH_4OH until pH = 7.0 was achieved. The mixture was extracted with dichloromethane (3 x 20 mL) and the extracts condensed in vacuo, then purified using flash column chromatography (1:99 MeOH:CH_2Cl_2) to produce **41** (0.29 g, 52%) as a pale yellow solid m.p:143 °C (dec). ^{1}HNMR (400 MHz, $CDCl_3$) δ 7.43 (t, 2H, *J*= 8.1 Hz), 7.30 (t, 1H, *J*= 7.3 Hz), 7.24 (t, 2H, *J*= 8.1 Hz), 6.99 (d, 1H, *J*= 8.1 Hz), 6.89 (d, 1H, *J*= 8.8 Hz), 3.86 (t, 4H, *J*= 5.1 Hz), 3.08 (t, 4H, *J*= 4.4 Hz). ^{13}CNMR (100 MHz, $CDCl_3$) δ 175.3, 159.5, 152.0, 150.2, 144.6, 140.2, 137.9, 129.9, 126.8, 121.6, 114.7, 112.6, 67.6, 52.1. LCMS found 371.9 $[M + H]^+$.

*Synthesis of 4-hydroxy-N-(4-hydroxy-7-morpholinobenzo[d]thiazol-2-yl)-4-methylpiperidine-1-carboxamide (**5b**).*[15b] Following the same method for the synthesis of compound **40**, compound **5b** was obtained as pale yellow solid (65 mg, 62%). m.p: 155 °C (dec). ^{1}H NMR (400 MHz, *d$_6$*-DMSO) δ 11.14 (brs, 1H), 9.36 (brs, 1H), 6.68-6.70 (m, 2H), 4.36 (brs, 1H), 3.83 (m, 2H), 3.73 (t, 4H, *J*= 4.41 Hz), 3.20-3.28 (m, 2H), 2.93 (t, 4H, *J*= 4.4 Hz), 1.36-1.48 (m, 4H), 1.12 (s, 3H). ^{13}CNMR (75 MHz, *d$_6$*-DMSO) δ 173.0, 155.0, 151.7, 139.0, 137.7, 112.7, 111.7, 108.0, 67.1, 66.5, 53.6, 52.0, 38.7, 30.2. LCMS found 393.0 $[M + H]^+$.

Bioassay procedures

Induction of liver injury

Mice were injected intravenously with Con A (20 mg kg^{-1}) in sterile PBS, and serum samples were taken or mice euthanized at indicated time points. Some mice were co-injected intraperitoneally with CGS21680 (2 mg kg^{-1}), **2** (2 mg kg^{-1}), **3** (2 mg kg^{-1}) and **4** (2 mg kg^{-1}) separately just before treatment with Con A. The magnitude or liver damage was evaluated by serum alanine aminotransferase (ALT) levels and liver tissue histology.

Measuring functionality of $A_{2A}R$ antagonism by cAMP assay

Stimulation of intracellular cAMP production and measurement of cAMP levels were performed as described previously.[18b] Lymphocytes were isolated from the spleen of C57/BL6 mice and suspended at a concentration of 400,000 cells/well, and then treated with 1 μM CGS 21680 **6** (A_{2A}R-specific agonist; from Tocris, Ellisville, MO) with or without KW-6002 **2**, KW-PEG **3**, preladenant **4**, tozadenant **5a** and their PEGylated analogs **27-31** and **40** (at a concentration of 1 mM/mL). The cells were incubated for 15 min at 37 °C, and the reaction was stopped by addition of 1 N hydrochloric acid. Levels of cAMP were determined by ELISA (Amersham Biosciences, Buckinghamshire, UK). All treatment groups were performed in triplicate.

Cytokine release assay

Splenocytes (2 x 10^6 /mL) were isolated from the spleen of C57/BL6 mice and activated with 0.1 μg/mL CD3 mAb to induce production of IFN-gamma. Immediately following the addition of mAb-CD3, the cells were treated with or without 1 μM CGS 21680 **6** agonist and 1 uM KW-6002 **2**, KW-PEG **3**, Preladenant **4** and compound **29**. Supernatants were collected after 24 h and levels of IFN-gamma were assayed by ELISA (Amersham Biosciences, Buckinghamshire, UK) using paired mAb and standard purchased from BD Pharmingen. All treatment groups were performed in triplicate.

Molecular modeling procedures

Homology modeling

The homology model from a previous report was employed for the docking study.[6] It was constructed via YASARA (Yet Another Scientific Artificial Reality Application) based on the crystal structure of A2AR in complex with ZM241385 **1** (PDB 3EML)[10] to fix the second extracellular loop (ECL2) residues Gln148 to Ser156. Residues were missing due to weak experimental electron density in that region. The quality of the homology model was examined by PROCHECK and verified as appropriate.

Computational studies

All calculations performed in this work were carried out on two Cooler Master Centurion 5 (Intel Core-i7 Quad CPU Q6600 @ 3.33GHz) operating systems running Maestro 10.4 (Schrödinger, LLC, New York, NY, 2015), ChemBioDraw® Ultra 14.0 (PerkinElmer) and YASARA. All pictures presented in this study were generated by Maestro and YASARA.

Molecule preparation

The ligands were prepared in ChemBio3D Ultra 14.0. The three-dimensional structures were built by importing the SMILES files of the ligands into LigPrep (LigPrep, version 3.6, Schrödinger, LLC, New York, NY, 2015), implemented in Maestro 10.4. LigPrep employs the Potentials for Liquid Simulations-all atom (OPLS-AA) force field 2005 for energy minimizations and a cellular pH value of 7.0 to generate the most probable ionization state of the ligand. [11d]

Docking studies

The homology model of $A_{2A}R$ was prepared using Maestro 10.4 Protein Preparation Wizard (Schrödinger, LLC, New York, NY, 2015). Before docking, bond orders were assigned and the orientation of hydroxyl groups, amide groups of the side chains of Asn and Gln, and the protonation state of His residues were optimized. A restrained refinement of the protein structure was performed using the default constraint of 0.3 Å RMSD and the OPLS 2005 force field. The docking studies were performed by Glide (Grid-Based Ligand Docking with Energetics) (Glide, version 10.4, Schrödinger, LLC, New York, NY, 2015). The enclosing docking box was set up with the centroid of four selected atoms, e.g. Glu169 (OE1), His250 (NE2), Asn253 (ND2) and His278 (NE2). The ligand diameter midpoint box was set up to be cubic with a box length of 20Å. No other constrains were applied. Both standard precision (SP) and extra precision (XP) docking protocols were carried out and the binding poses were examined.

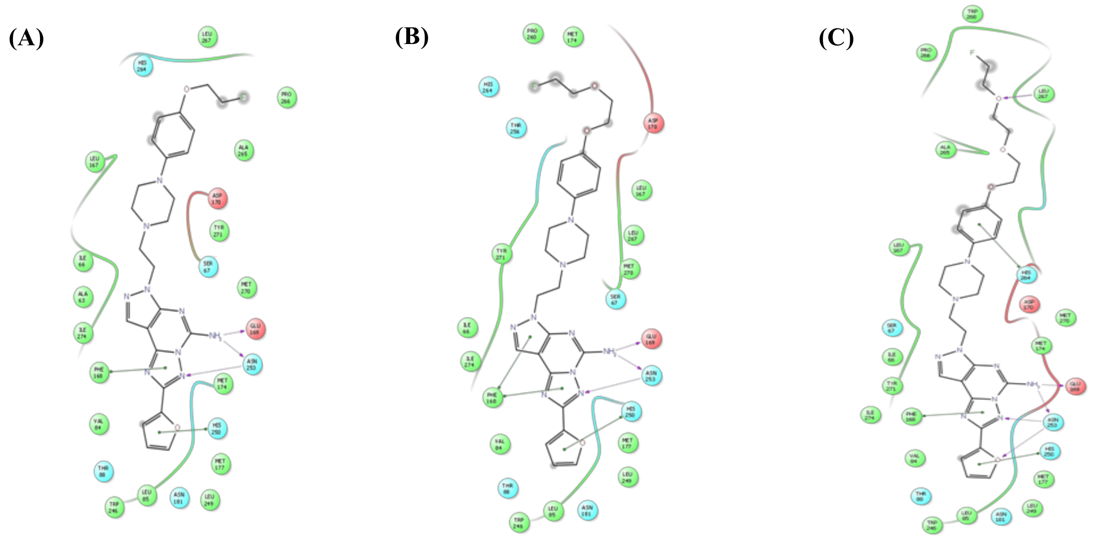

Figure S1. Docking results of compounds **27-29** via Glide XP method. The H-bond interaction is represented as a pink arrow, pi-pi stacking as a green arrow. (A) Compound **27** docking pattern. (B) Compound **28** docking pose. (C) Compound **29** docking result. Pictures are rendered from Glide.

Determination of Physicochemical Properties

Aqueous pH 7.4 Solubility

Compounds were dried down from 10 mM DMSO solutions using centrifugal evaporation techniques. Phosphate buffer (0.1 M pH 7.4) was added and StirStix inserted in the glass vials. Agitation was then performed at a constant temperature of 25°C for 20-24 hours. The vials were then subjected to double centrifugation (with a tip wash in between) to eliminate any residues of the dried compound. The solutions were then diluted and quantitated using LC/MS/MS.

Log $D_{7.4}$

A shake-flask method was employed to determine octanol-water distribution coefficients at pH 7.4 (Log $D_{7.4}$). The aqueous solution used was 10 mM sodium phosphate pH 7.4 buffer. The method was validated for Log $D_{7.4}$ ranging from -2 to 5.0.

Human Plasma Protein Binding (PPB)

PPB was determined using equilibrium dialysis (RED device) to separate free from bound compound. The amount of compound in plasma (10 µM initial concentration) and in dialysis

buffer (pH 7.4 phosphate buffer) was measured by LC-MS/MS after equilibration at 37°C in a dialysis chamber. The fraction unbound (fu) is reported.

Intrinsic clearance

In vitro intrinsic clearance was determined from human liver microsomes or rat hepatocytes using standard methods. Following incubation and preparation, the samples were analyzed using LC/MS/MS. Refined data were uploaded to IBIS and displayed as CL_{int} (intrinsic clearance) in μl/min/mg or μl/min/1 million cells.
